# Cross platform microarray analysis for robust identification of differentially expressed genes

**DOI:** 10.1186/1471-2105-8-S1-S5

**Published:** 2007-03-08

**Authors:** Roberta Bosotti, Giuseppe Locatelli, Sandra Healy, Emanuela Scacheri, Luca Sartori, Ciro Mercurio, Raffaele Calogero, Antonella Isacchi

**Affiliations:** 1 Genomics Unit, Biotechnology Department, Nerviano Medical Sciences S.r.l., Viale Pasteur 10, 20014 Nerviano (MI), Italy; 2 Research Informatics Group, Chemistry Department, Nerviano Medical Sciences S.r.l., Viale Pasteur 10, 20014 Nerviano (MI), Italy; 3 Genomics and Bioinformatics Unit, Dipartimento di Scienze Cliniche e Biologiche c/o Az. Ospedaliera S. Luigi Regione Gonzole 10, 10043 Orbassano (TO), Italy; 4 Current address: Genextra S.p.A. c/o IFOM-IEO Campus, Via Adamello 16, 20134 Milan, Italy

## Abstract

**Background:**

Microarrays have been widely used for the analysis of gene expression and several commercial platforms are available. The combined use of multiple platforms can overcome the inherent biases of each approach, and may represent an alternative that is complementary to RT-PCR for identification of the more robust changes in gene expression profiles.

In this paper, we combined statistical and functional analysis for the cross platform validation of two oligonucleotide-based technologies, Affymetrix (AFFX) and Applied Biosystems (ABI), and for the identification of differentially expressed genes.

**Results:**

In this study, we analysed differentially expressed genes after treatment of an ovarian carcinoma cell line with a cell cycle inhibitor. Treated versus control RNA was analysed for expression of 16425 genes represented on both platforms.

We assessed reproducibility between replicates for each platform using CAT plots, and we found it high for both, with better scores for AFFX. We then applied integrative correlation analysis to assess reproducibility of gene expression patterns across studies, bypassing the need for normalizing expression measurements across platforms. We identified 930 genes as differentially expressed on AFFX and 908 on ABI, with ~80% common to both platforms. Despite the different absolute values, the range of intensities of the differentially expressed genes detected by each platform was similar. ABI showed a slightly higher dynamic range in FC values, which might be associated with its detection system. 62/66 genes identified as differentially expressed by Microarray were confirmed by RT-PCR.

**Conclusion:**

In this study we present a cross-platform validation of two oligonucleotide-based technologies, AFFX and ABI. We found good reproducibility between replicates, and showed that both platforms can be used to select differentially expressed genes with substantial agreement. Pathway analysis of the affected functions identified themes well in agreement with those expected for a cell cycle inhibitor, suggesting that this procedure is appropriate to facilitate the identification of biologically relevant signatures associated with compound treatment. The high rate of confirmation found for both common and platform-specific genes suggests that the combination of platforms may overcome biases related to probe design and technical features, thereby accelerating the identification of trustworthy differentially expressed genes.

## Background

Potential applications of genomics in Oncology cover the whole spectrum of pathology, diagnosis and treatment. Microarrays, usually in combination with Quantitative Real Time PCR (RT-PCR), are emerging as the method of choice for genome-scale gene expression analysis and several commercial platforms are currently available.

In the past few years a tremendous effort has been made, in the academic, pharmaceutical and clinical community, to better understand oncogenic processes, to develop innovative drugs targeted to the molecular lesions underlying specific cancer subtypes, and to identify the patient population that can best benefit from the new therapies [1-4]. This effort requires the integrated use of data across multiple laboratories, to link cancer biology to the mechanism of action of the new drugs, and finally to translate the preclinical findings into the proof of concept of target modulation in patients.

During the preclinical phase of drug development, lead profiling with microarrays can help to identify the intracellular pathways that are perturbed by each chemical compound, contributing to a better understanding of its mechanism of action and possible side effects, and potentially leading to the identification of a gene signature correlated with efficacy or safety [5-8]. For this purpose, the lead compounds are typically analyzed in dose response and time course experiments for their ability to modulate gene expression in tumor cell lines tested in vitro and in vivo. The comparison of these data with results on gene expression profiling of different tumors can also contribute to the identification of the tumor types that can respond better to the drug.

Despite the rapid progress in the field, many important aspects, including the reproducibility, reliability and standardization of microarray analysis and results will have to be addressed before the routine application of microarray data in the clinic.

While the multiplicity of microarray platforms offers an opportunity to expand the use of the methodology and make it more easily available to different laboratories, the comparison and integration of data sets obtained with different microarray platforms is still challenging [9-21]. Sources of diversity arise from the technology features intrinsic to chip manufacturing, from the protocols used for sample processing and hybridization, from detection systems, as well as from approaches applied to data analysis. On one hand, the combined use of multiple platforms can overcome the inherent biases of each approach, and may represent an alternative that is complementary to RT-PCR for identification of the more robust changes in the gene expression profiles. On the other hand, the comparison of data generated using different platforms may represent a significant challenge, particularly when considering very different systems (one vs. two channel approach, cDNA vs. oligo-based chip).

In this paper, we combined statistical and functional data analysis for the cross platform validation of two oligonucleotide-based technologies, Affymetrix GeneChip^® ^(AFFX) [22] and Applied Biosystems Human Genome Survey Microarrays^® ^v. 1.0 (ABI) [23], and validated the results with RT-PCR.

AFFX is a well known technology characterized by in situ synthesized 25 mer oligonucleotides, that uses fluoresence as the detection system. ABI is a recently introduced technology based on nylon-spotted 60 mer oligonucleotides, that uses one oligo to detect each gene for most genes, chemiluminescence to measure gene expression levels and fluorescence to grid, normalize and identify microarray features. The ABI gene list combines information from public and Celera databases.

The choice for these two platforms was based on the idea of comparing a widespread microarray technology with a more recent long oligonucleotide-based platform that also uses a single colour channel, but with a different detection system.

In order to test the platform performance under conditions close to our most common experimental settings, we analyzed the effects of drug treatment with a cell cycle inhibitor compound that had been previously characterized for mechanism of action and activity in tumor cells. While both microarray platforms performed well individually, we developed a robust cross-platform analysis pipeline and showed that it can be applied to accelerate the identification of trustworthy differentially expressed genes.

## Results

**Figure 1:**
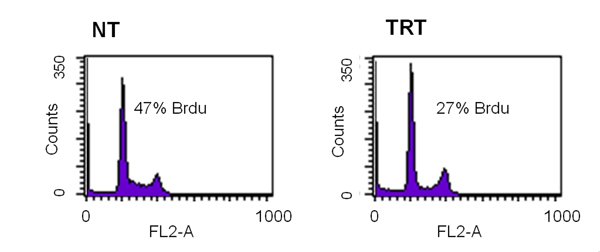
**FACS analysis**. A2780 cells were left untreated (NT) or treated for 6 hours with a cell cycle inhibitor (TRT). BrdU was added 30 min before harvesting and samples were processed for cell cycle analysis and BrdU incorporation analysis.

In this study, we analysed differentially expressed genes after a 6 hour treatment of the ovarian cancer cell line A2780 with a cell cycle inhibitor. The activity of the compound was confirmed by FACS analysis where an accumulation of cells in G1 phase of cell cycle was observed, associated with a reduction in DNA duplication as measured by a decrease in BrdU incorporation (Figure 1). Total RNA from treated and control samples was processed according to the manufacturers' recommended protocols, divided in three technical replicates for each platform and hybridised to AFFX HGU133plus2 or ABI Human Genome Survey Microarray v.1.0.

All data from this study were uploaded to National Center for Biotechnology Information, Gene Expression Omnibus [24] with the GSE ID GSE6140

### Intraplatform reproducibility

We used the new descriptive CAT (Correspondence At the Top) plots originally proposed by Irizzarry [9] to evaluate the array-to-array precision within each microarray platform for the three replicates. This method addresses the issue of array-to-array comparison within the same platform under "normal" conditions, in which we expect only a small subset of genes to be differentially expressed. As described by Irizzarry, genes characterized by log_2_(Fold Change) close to zero are probably not differentially expressed and they may not show a good correlation between platforms or experimental replicates. Therefore it is more important to assess agreement for genes that show significant log_2_(Fold Change) between treatments in order to evaluate the agreement between experimental replicates.

**Figure 2:**
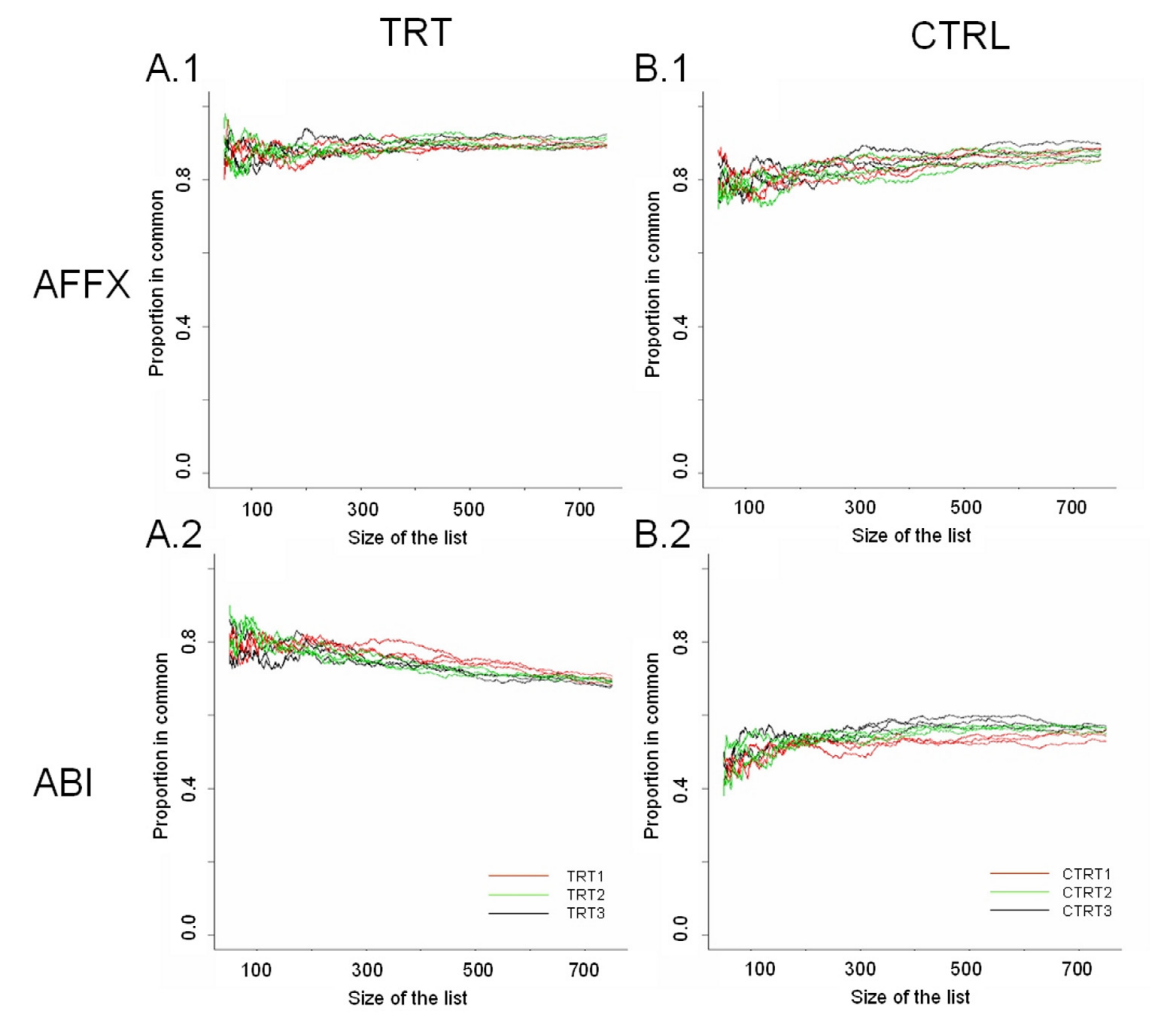
**Intraplatform reproducibility**. The descriptive CAT (Correspondence At the Top) plots [9] were used to evaluate the array-to-array precision within each microarray platform for the three replicates. CAT Plots describe the proportion of genes in common between replicates as function of list size. To generate CAT Plots on treated samples we used the lists of genes ranked by |log_2_(fold change)| created by each of the treated samples (TRT) versus a reference, which is one of the control samples (CTRL), both for AFFX (A.1) and ABI (A.2). Similarly we calculated CAT Plots for CTRL samples using one of the TRTs as reference for AFFX (B.1) and ABI (B.2).

To focus the comparison on the genes that appeared to be more differentially expressed across technical replicates, we compared two technical replicates at a time, and generated lists of genes of increasing size, up to 700, ordered from high to lower log_2_(Fold Change). We then generated a CAT plot to analyze the consistency of the lists (Figure 2).

In the AFFX platform all controls as well as the treated replicates are very homogeneous as shown by the strong overlap between sample specific curves (Figure 2A.1 and 2B.1). Comparable results were obtained for ABI data on treated samples (Figure 2A.2), while the quality of "correspondence at the top" is less consistent for the control samples (Figure 2B.2), which might reflect a higher degree of baseline data variability. It should be noted that the ABI Human Genome Survey Microarray v. 1.0 used in this experiment are not the most updated version available. For completion, we also evaluated R squared values between the various replicates, which were >0.99 for AFFX, and >0.94 for ABI, both in control and treated samples. The difference we noticed with CAT plots on ABI controls is less evident by R-square calculation, reflecting the higher sensitivity of CAT plots. These results suggest that both platforms have overall good reproducibility across technical replicates.

### Interplatform reproducibility

In order to assess differences/similarities between the two platforms, only genes common to both platforms were used in the comparison. Transcripts represented on both platforms were identified using Resourcer [25], and Entrez Gene ID was used as common identifier.

**Figure 3:**
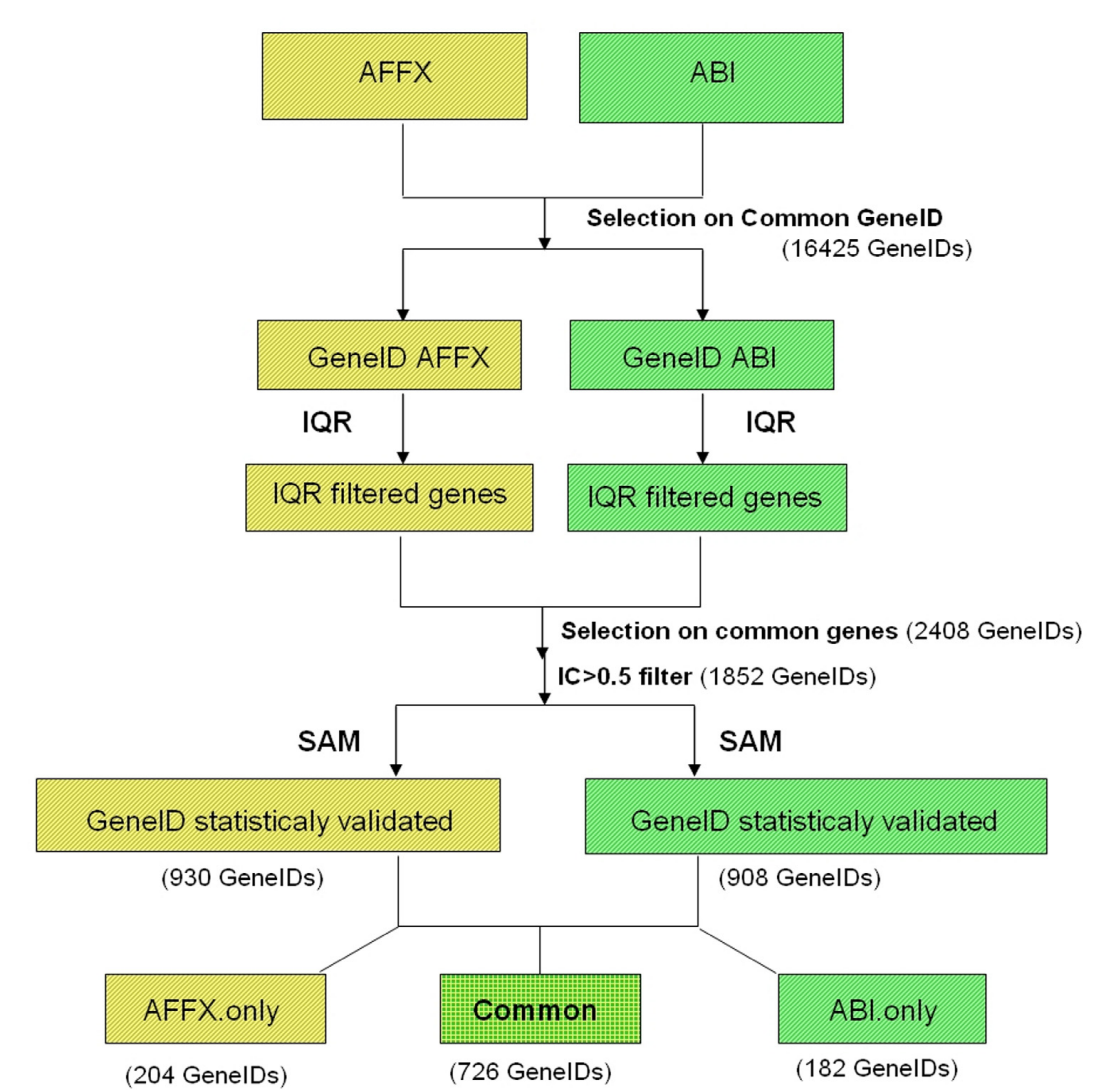
**Microarray analysis Pipeline**. Microarray analysis pipeline applied to AFFX and ABI data to identify lists of differentially expressed genes.

AFFX and ABI data were processed independently but the same procedure was applied (Figure 3). Differential expression ratios log_2_(Fold Change) were compared between platforms to define cross platform correlation. When log_2_(Fold Change) relative to all 16425 genes were compared between platforms, the correlation was weak (r = 0.53, where "r" represents the Pearson correlation coefficient).

However, when we applied a filtering step [26] in order to remove genes showing little variation across samples (IQR < 0.4), the correlation between microarray platforms improved (r = 0.68), in agreement with results reported in other cross-platform comparison studies that show the importance of filtering data prior to further analysis [12,17].

For the subset of 2408 genes common to both platforms after the filtering procedure, we calculated the Integrative Correlation (IC) coefficient according to Parmigiani [27]. Since AFFX and ABI use different technologies to measure transcript expression, the absolute signal values for each platform tends to be somewhat arbitrary and not suitable for correlation analysis across platforms. Integrative correlation is a systematic statistical approach based on linear correlations that allows assessment of the reproducibility of gene expression patterns across studies, bypassing the need for normalizing expression measurements across platforms. Consistency of gene coexpression patterns would reflect the overall consistency of the data sets. To perform this analysis, each of the possible gene-to-gene correlations was calculated within each platform, and these correlations were then compared across the two platforms.

To identify statistically significant differentially expressed genes we used only 1852 genes characterized by an IC > 0.5 across the two platforms. Limiting the comparison to these subgroups, the correlation improves further to 0.80.

**Figure 4:**
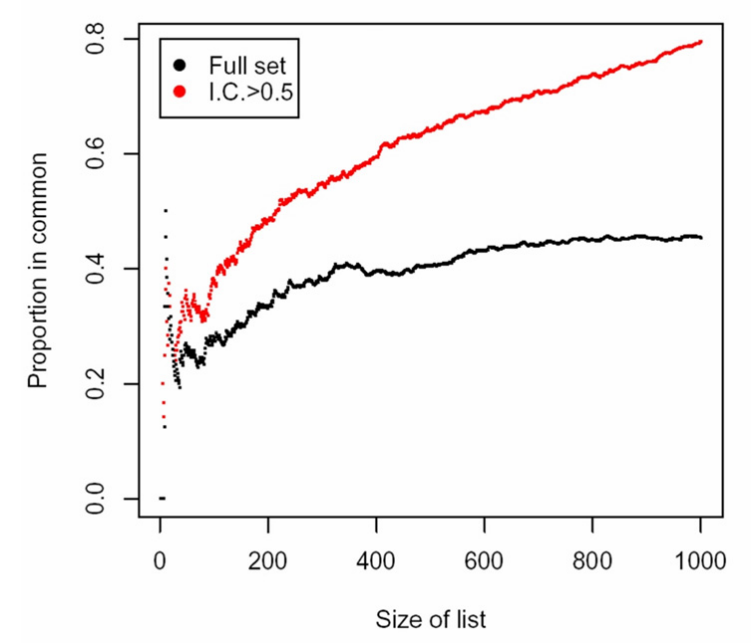
**Interplatform agreement**. The descriptive CAT (Correspondence At the Top) plots [9] were used to evaluate interplatform agreement. The Correspondence at the top was evaluated for AFFX and ABI using the full set of genes after the IQR filtering step (black line) or after the IC filtering step (red line). For each of the two platforms average |log2 (Fold Change)|, calculated between treatment vs. control group, was used to generate CAT Plots.

We then used CAT plots [9] to show that the quality of differential expression similarity between the two platforms increases when only the 1852 genes with IC > 0.5 are used, compared to the 2408 subset (Figure 4). If the list of 2408 genes is used the agreement ranges between 35% to 40% for lists greater than 400 genes. The use of the subset of 1852 genes derived by the IC filtering improves the agreement to over 60%. Statistical validation performed using "Significant analysis of microarrays" (SAM) [28], allowed the identification of 930 differentially expressed genes for AFFX and 908 for ABI. Interestingly, 726 genes (~80% in both cases) were identified as differentially expressed in both platforms, with 204 unique to AFFX and 182 unique to ABI (Figure 3).

**Figure 5:**
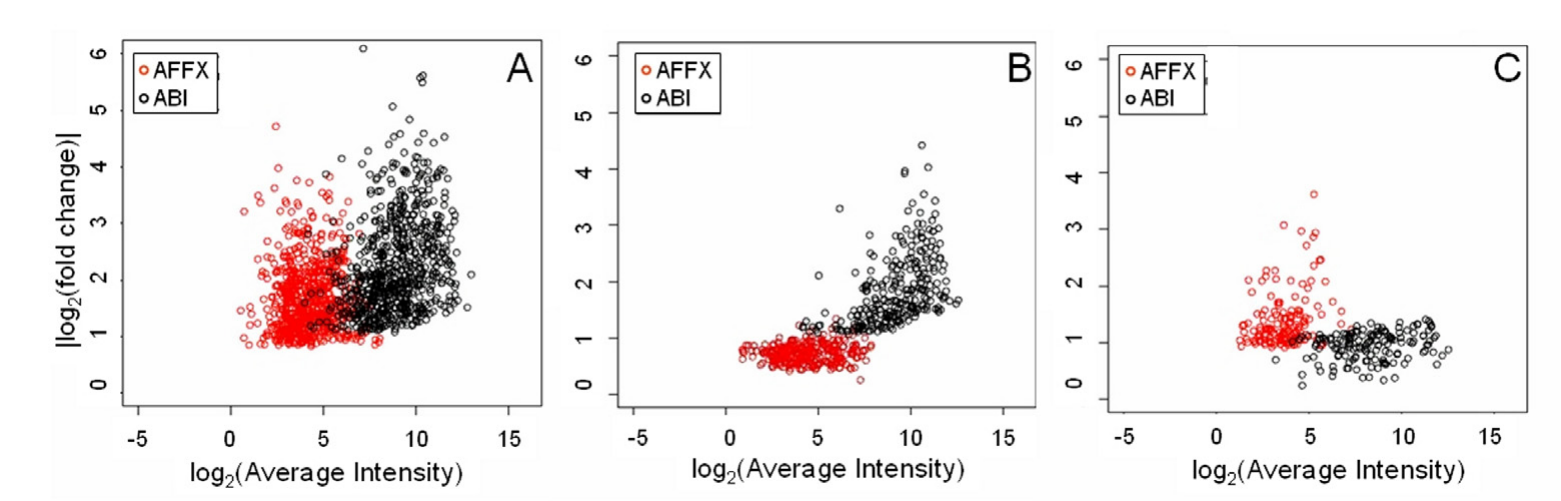
**Genes found differentially expressed using AFFX and ABI**. A) Set of 726 genes passing SAM statistical validation both in AFFX and ABI, B) Set of 182 genes passing SAM statistical validation only in ABI, C) Set of 204 genes passing SAM statistical validation only in AFFX. AFFX data are reported in red, ABI in black. The lack of statistical significance in one of the platforms (B, C) is also associated with a limited log_2_(Fold Change) variation. On the other hand, log_2_(Fold Change) variation for genes passing the statistical validation in both platforms (A) is quite similar, although ABI seems to have wider log_2_(Fold Change) dynamic range.

We then analyzed in parallel the spread of log_2_(Fold Change) and log_2_(Average Intensity) values for AFFX and ABI for the common genes, as well as for the unique subsets (Figure 5). Despite the different absolute values, the range of intensities of the differentially expressed genes detected by each platform was similar. ABI showed a slightly higher dynamic range in the FC values, which might be associated with its original detection system. Similar results were also obtained for genes detected only by each single platform.

### Validation of measurements for shared and unique expression profiles

RT-PCR is often referred to as the "gold standard" for gene expression measurements [11,19,29], due to its advantages in detection sensitivity, sequence specificity, large dynamic range, as well as its high precision and reproducible quantitation compared to other techniques [30-32]. For these reasons, we used RT-PCR for independent validation of microarray results.

RT-PCR was performed on 66 genes, including a subset of 26 genes out of the 726 common to both platforms, 19 genes detected only by AFFX and 21 genes detected only by ABI. Importantly, genes for validation were randomly chosen to represent the whole range of intensity signals and FC differences. It's worth mentioning that primers were selected without referring to the position of AFFX or ABI probes. Indeed this design allowed us to validate the actual expression of each gene and not simply the signal detected by microarray that usually has probes designed in the 3' UTR region.

All genes detected as differentially expressed by both microarray platforms were also found to be differentially expressed by RT-PCR (pValue < 0.5), although differences in the magnitudes of individual expression ratios were observed (Additional File 1). Interestingly, all genes detected by one platform but not the other were also confirmed to be differentially expressed, the only exceptions being 4 cases in which the RT-PCR results were not technically acceptable (36/40), suggesting that the combined usage of two platforms might allow the detection of a subset of truly differentially expressed genes that would have been lost if only one platform was used. The overall confirmation rate (62/66) is particularly interesting since the genes were chosen to span the whole range of intensity and fold change values of microarray data.

To explain the subsets of specific genes detected by the two platforms, we evaluated: i) GC content of AFFX and ABI probes, ii) gene location of the probes and iii) presence of highly stable secondary structure in the mRNA region involved in the hybridization. However, these characteristics were comparable across the common and unique datasets, suggesting that other parameters such as hybridization kinetics, steric hindrance of probe hybridization, method of detection and others, might be involved [15].

It was recently suggested that the reorganization of AFFX probes into gene specific probe sets may help to generate more accurate information, resulting ultimately in a better interpretation of the data [33]. Dai *et al*. applied a series of probe selection and grouping criteria to generate new GeneChip library files (hereafter called custom CDF) according to different target definitions, such as UniGene, Refseq, ENSEMBL Entrez Gene, etc. In order to verify if the use of Entez Genes (GeneID) custom CDFs could improve the concordance between the two platforms, we extracted the corresponding set of AFFX probes, validated with RT-PCR, from these custom CDF.

Only 18 out of 62 validated genes were mapped with this gene-oriented approach on custom CDF (data not shown), suggesting that Entrez Gene based CDFs, although designed to be more target specific, result in loss of significant differential expression. However, more transcript-oriented custom CDFs, i.e. RefSeq, might overcome this problem.

### Theme enrichment

There is increasing evidence that even if the exact list of differentially expressed genes that are identified using different platforms overlap only partially, the biological themes represented by these genes are the same [34]. Based on this we investigated the level of concordance of biological themes represented in the data across the two platforms using Ingenuity Pathways Analysis (IPA) 3.1 software (Ingenuity^® ^Systems) [35], a commercial database containing manually annotated data for human protein-protein and functional interactions derived from the literature. The set of genes in common between AFFX and ABI recapitulates the themes related to cell cycle control, cell proliferation and differentiation and DNA replication (Additional File 2). These themes fit the expected functional effect linked to a cell cycle inhibitor. The same themes were also found for the platform specific genes. In addition, a few functions not represented in the common subset were also identified (Additional File 2), supporting the concept that the integrated use of more than one platform can amplify the ability to detect biologically relevant genes that are affected by treatment.

## Discussion

A series of studies have been reported on evaluating performance across various commercial and homemade microarray platforms, with contradictory results. A number of groups have reported limited concordance of results across expression analysis platforms [13,17,21,36-39]. However, recent publications have reached more positive conclusions about the possibility of comparing data, reinforcing the emerging concept that data treatment and choice of comparison metric plays a fundamental role in this approach [9,10,15,40].

In the past few years, AFFX has been analysed in parallel with many other platforms, as a widespread technology that can be used as a reference standard.

Barnes et al. [15] published a comparison of AFFX with the Illumina, a recently introduced long-oligonucleotide bead-based array, where, despite the fundamental technical differences of the two approaches, they reported a very high agreement of results, particularly once the factors of gene expression level and probe placement on the gene are considered. In particular, they found that expression level plays a major role in determining reproducibility across platforms, and that the precise location of the probe on the genome affects the measurement to a substantial degree. Irizzarry et al. [9] also reported a relatively good agreement between AFFX and two-color systems, and raised the important points that absolute measurements of gene expression cannot be used to assess data across platforms (both studies using absolute measurements had found disagreement [13,36]) and that data pre-processing has significant effects on final results.

In this study, we have analysed the performance of the AFFX and ABI platforms in parallel on the same sample. To put ourselves in conditions close to a "real world" experiment, we analysed technical replicates of a control vs. treated sample, in which we used a cell cycle inhibitor that we had previously characterized in biochemical and cell-based assays. While many comparisons between oligonucleotide arrays have been carried out in the past, as already discussed [12,13,21,41], to our knowledge this study is the first to examine the comparison between AFFX and ABI. Recently, a large-scale real-time validation experiment was published, where results from ABI and Agilent Whole Human Genome Oligo Microarrays^® ^were confirmed in parallel by RT-PCR [19] showing a reasonable coherence between the two types of data for both platforms, with good sensitivity, while the specificity of microarray data tended to be relatively low, in particular for Agilent.

While many authors underline the importance of verifying microarray results using RT-PCR as a reliable independent technology for gene expression measurement, this approach is not always straightforward, since it is expensive and time consuming, usually only allowing the reconfirmation of a very small fraction of the results.

Barnes et al. [15], in their comparison of AFFX and Illumina platform already noticed how, in contrast to studies where few results are checked by RT-PCR, the use of two combined platforms can be considered as a built-in cross validation of a huge fraction of the results of the experiment. Our results strongly suggest that the use of an approach based on two single channel microarray platforms combined with an analytical pipeline as applied here, can achieve this objective. Indeed the confirmation rate we obtained of 62/66 genes is particularly good, taking into account that these genes were selected from the list of differentially expressed with the aim of covering the whole range of log_2_(Fold Change) and Average Intensity values observed in each platform. This approach seems more effective for the identification of truly differentially expressed genes than theoretical approaches, such as the use of a more robust annotation, like custom CDF [33], that in our hands resulted in loss of 44 out of 62 experimentally validated genes.

We have found that the critical point for a trustworthy identification of differentially expressed genes is the availability of methods that measure the correlation/similarity between transcription profiles generated with different platforms. Meta-analysis tools and strategies for combining data from microarray experiments have been proposed [27,42]. Among these, integrative correlation is a tool that, assessing overall reproducibility of gene co-expression patterns across studies, can possibly be used to identify genes with relatively consistent co-regulation patterns. The strength of our pipeline is the use of the integrative correlation coefficient, since this is the filter that removes uncorrelated profiles between the two platforms. This may also explain the high degree of RT-PCR confirmation that we also observed for the unique subset of genes that were identified as differentially expressed by only one of the two platforms.

A complementary way to assess the soundness of our approach is the compatibility of the results with the expected data, based on the previous knowledge of the mechanism of action of the compound. The set of genes in common between AFFX and ABI were analyzed with Ingenuity^® ^Systems [35] to detect theme enrichment and were shown to recapitulate themes that fit well with the expected functional effect linked to a cell cycle inhibitor (including cell cycle, cell death, cell signaling, cellular growth and proliferation and DNA replication). Furthermore, the coherence of biological themes identified even within the platform specific gene list suggests that this cross platform analysis could enhance the biological information that can be gained from microarray data.

Since there is no fundamental difference in the common vs. unique subset of genes as far as the range of their intensity and log_2_(Fold Change) values is concerned, we looked for an alternative explanation for the lack of recognition by one of the two platforms. Although we have investigated GC content, probe position and secondary structure effects of the target, none of them was conclusive. It has to be noted that although the genes in these unique subsets did not pass the statistical analysis, in many cases they were found to be differentially expressed with borderline log_2_(Fold Change) values, reinforcing the overall good comparability of data across the two platforms.

## Conclusion

In this study we present a cross-platform validation of two oligonucleotide-based technologies, Affymetrix GeneChip^® ^and Applied Biosystems Human Genome Survey Microarrays^® ^v. 1.0. For both platforms, we found good reproducibility between technical replicates, and showed that both platforms can be used to select differentially expressed genes with substantial agreement. 62/66 selected genes were confirmed by RT-PCR as being differentially expressed. Pathway analysis of the affected functions identified themes well in agreement with those expected for a cell cycle inhibitor, suggesting that this procedure is appropriate to facilitate the identification of biologically relevant signatures associated with compound treatment. The high rate of confirmation found for both common and platform-specific genes suggests that the combination of two platforms may overcome biases related to probe design and technical features intrinsic to individual systems, thereby expanding the ability to identify truly differentially expressed genes.

## Methods

### RNA preparation

Human Ovarian cell line A2780 [43,44] was obtained from ECACC (Cat no.93112519) and cells were untreated or exposed to 3 μM of a cell cycle inhibitor for 6 hrs. Three biological replicates were performed for each treatment. Only attached cells were harvested. RNA was purified using a Qiagen RNA purification kit. RNA was quantitated using a spectrophotometer and the quality of the RNA was assessed using a Bioanalyser.

Biological replicates were pooled to obtain a unique sample for each treatment, which was then divided to generate three aliquots for each condition (technical replicates) per platform.

### Affymetrix array experimental procedure

The experiment was performed at IFOM Affymetrix Facility (IFOM-IEO Campus, Milan, Italy).

5 μg of each RNA pool was used for the amplification/labelling reaction. 1^st ^and 2^nd ^strand cDNA synthesis was performed with the Invitrogen kit (11917-020) and the IVT reaction was done with Megascript T7 from Ambion (1334). All steps were done according to manufacturers instructions [22] and cRNA was quantitated on a spectrophotometer. 15 μg of cRNA was fragmented and checked by denaturing gel electrophoresis. Bacterial transcripts at the cRNA level were spiked into each sample prior to hybridisation. 10 μg of fragmented cRNA was hybridised to a Human Genome U133 Plus 2.0 Array (900466).

Three technical replicates of each sample were performed. Hybridisations, washing and staining were performed according to AFFX protocols. Hybridised arrays were scanned with the Genechip Scanner 3000. Probe set intensities were calculated using the RMA algorithm [45] and normalized by the quantiles method [46,47].

### Applied biosystem procedure

The experiment was performed at Genesys Applied Biosystem Facility (Genesys, Munster, Germany).

2 μg of each RNA pool was labeled with Digoxigenin-UTP using the ABI Chemiluminescent RT-IVT Labeling Kit v 1.0 accordingly to manufacturer's protocol [19]. 10 μg of the labeled cRNA was hybridized to ABI Human Genome Survey Microarray v 1.0. Following hybridization and washing steps, chemiluminescent detection and image acquisition was performed using Applied Biosystems 1700 Chemiluminescent Microarrays Analyzer, following manufacturer's protocol. For inter-array normalization, a global median normalization was applied across all microarrays. Normalized expression levels were imported as exprset in Bioconductor [48].

### Cross-mapping between microarrays platforms

Transcripts present on both platforms were identified using Resourcer [25], and Entrez Gene ID was used as a common identifier.

At the time of the analysis 54675 probe sets of AFFX HGU133plus2.0 GeneChip where mapped to 18857 unique GeneID, while 33096 probes of ABI Human Genome Survey Microarray v. 1.0 where linked to 17109 unique GeneID. The two platforms shared a total of 16425 GeneID.

AFFX has high redundancy in Probe sets, ABI also has some redundancy but to a lesser extent. Therefore, when more than one probe set/probe exists for the same Gene ID, we selected as representative for that Gene ID the probe set/probe with the lowest p-value in a t-test analysis. If two or more probes have the same p-value, that with the highest log_2_(Fold Change) was chosen. We applied these criteria both to AFFX and ABI data.

### Data analysis

Microarray data analysis was performed using Bioconductor libraries [48]. AFFX and ABI data sets were filtered to select probe sets with an intra-experiment Inter Quantile Range (IQR) less than 0.4. AFFX and ABI data were processed independently but using the same procedure (Figure 3). Selecting the 2408 common genes, we used integrative correlation analysis (IC) [27] to assess overall reproducibility of gene coexpression patterns across the two platforms and to identify genes with relatively consistent coregulation patterns. Within each study, and for each pair of genes, we calculated the correlation coefficient of expression values across subjects. By examining whether, for a specific gene, these correlations agree across studies we can quantify the reproducibility of results without relying on direct comparison of expression across platforms. The IC provides a reproducibility score for each gene. This analysis is unsupervised in that consistency is measured without using information about sample phenotypes. To identify statistically significant differentially expressed genes we used only 1852 genes characterized by an IC > 0.5 across the two platforms.

Subsequently, SAM [28] implemented in Bioconductor libraries was used to identify probe sets differentially expressed between compound treatment and control. Differentially expressed genes were identified with a two-class unpaired method. A threshold value can be adjusted to maximize the number of significant genes while minimizing the predicted false discovery rate. We conducted a blocked, two-class unpaired test using a threshold allowing a false significant number of about 0.3. This analysis produced 930 differentially expressed genes for AFFX and 908 for ABI. 726 genes (~80%) were identified as differentially expressed in both platforms, with 204 unique to AFFX and 182 unique to ABI (Figure 5).

### Functional analysis

Themes enrichment and pathway analysis was performed using Ingenuity Pathways Analysis (IPA) 3.1 software (Ingenuity^® ^Systems) [35], a commercial database containing manually annotated data for human protein-protein and functional interactions derived from the literature.

### Real-time QPCR

Total RNA was reverse-transcribed using Applied Biosystems Reverse Transcription kit following manufacturers instructions in a 25 μl reaction volume; resulting cDNA was diluted in TE buffer to a final concentration of 5 mg/ml, prior to PCR amplification using Applied Biosystems "real time" version of the assay on the ABI Prism 7900 thermal-cycler. RT- PCR was done using Applied Biosystems Sybr green Master Mix 1x, primers 300 nM, 12.5 ng of cDNA in 12.5 μl of reaction volume; the reaction began with 10 minutes at 95°C, followed by 40 cycles of 15 seconds at 95°C and 45 seconds at 60°C.

PCR oligonucleotide primers were selected to specifically amplify fragments of selected human genes using the freely available Primer3 [49], and were synthesized in the in house facility; complete gene sequences were downloaded from GeneBank NCBI website and specificity of primers, whose sequence was designed in correspondence with the exon junctions conserved in all known alternative spliced forms, was checked using NCBI BLAST [50].

The analysis of RT-PCR output data followed the manufacturer-suggested ΔΔCt method, that provides the target gene expression value as unitless fold changes in the unknown sample compared to a calibrator sample; both unknown and calibrator sample target gene expressions are normalized by the relative expression of housekeeping genes (18S RNA gene, beta-actin, cyclophilin A, beta-glucoronidase).

The calibrator sample was obtained by reverse-transcription of a mix of the twelve human tissue RNA contained in the Clontech RNA Panel I and IV.

Statistical evaluation of treatment comparison has been performed by t-test analysis using Spotfire DecisionSite^® ^8.0 [51].

## Authors' contributions

RB performed analysis of the data, generated all the figures and drafted and finalized the manuscript. GL prepared the samples and helped with microarray experiment, conducted and analyzed the RT-PCR data and contributed to the general organization of the experiment. SH assisted with the microarray experiment that generated the data and contributed to the pathway analysis, discussion and manuscript revisions. ES performed pathway analysis and contributed to discussion. LS helped with analysis, read the manuscript and provided comments. CM helped with experiment planning and actively contributed to discussion. RC helped with data analysis and wrote scripts for data parsing, providing overall technical guidance and coordination. AI planned and designed the experiment. RC and AI supervised and coordinated the project and assisted with the interpretation. All authors have read and approved the manuscript.

## Supplementary Material

Additional File 1 **RT-PCR validation of selected genes**. RT-PCR validation of selected genes. Fold Change values are reported. Click here for file

Additional File 2 **Themes enrichment detected using IPA 3.1 software (Ingenuity^® ^Systems)**. Themes enrichment detected using IPA 3.1 software (Ingenuity^® ^Systems). Click here for file
